# Unraveling the Genomic Association for Milk Production Traits and Signatures of Selection of Cattle in a Harsh Tropical Environment

**DOI:** 10.3390/biology12121483

**Published:** 2023-12-02

**Authors:** Silpa Mullakkalparambil Velayudhan, Tong Yin, Shahin Alam, Kerstin Brügemann, Veerasamy Sejian, Raghavendra Bhatta, Eva Schlecht, Sven König

**Affiliations:** 1 Institute of Animal Breeding and Genetics, Justus-Liebig-University Gießen, Ludwigstraße 21 b, 35390 Gießen, Germany; mv.silpa@gmail.com (S.M.V.); tong.yin@agrar.uni-giessen.de (T.Y.);; 2 Animal Husbandry in the Tropics and Subtropics, University of Kassel and Georg-August-Universität Göttingen, Steinstr. 19, 37213 Witzenhausen, Germany; shahindps@uni-kassel.de (S.A.);; 3 National Institute of Animal Nutrition and Physiology (NIANP), Hosur Rd, Chennakeshava Nagar, Adugodi, Bengaluru 560030, India

**Keywords:** challenging environment, dairy cows, GWAS, milk traits, mastitis, selection signature, thermotolerance

## Abstract

**Simple Summary:**

Genomic association analyses for milk yield and milk quality traits including mastitis were accomplished in a dairy cattle population from the harsh and challenging tropical savanna climate of Bengaluru, India. A further effect of selection was due to social–ecological drivers, i.e., specific constraints that are prevalent in small-sized family farms. As a time report, this study identified footprints of selection among the dairy cattle breeds in this region. The genome-wide association study (GWAS) identified two SNPs, rs109340659 and rs41571523, significantly associated with test-day milk yield. Fibrosin-like 1 (*FBRSL*) and calcium voltage-gated channel auxiliary subunit gamma 3 (*CACN*) were the respective annotated potential candidate genes. Furthermore, the genomic regions under selection were associated with pathways and mechanisms involving ubiquitination, cell signaling and immune response. Hence, contrasting dairy breeds genomically contributed to the detection of genomic regions under selection, with strong effects on adaptation and overall disease resistance.

**Abstract:**

A study was designed to identify the genomic regions associated with milk production traits in a dairy cattle population reared by smallholder farmers in the harsh and challenging tropical savanna climate of Bengaluru, India. This study is a first-of-its-kind attempt to identify the selection sweeps for the dairy cattle breeds reared in such an environment. Two hundred forty lactating dairy cows reared by 68 farmers across the rural–urban transiting regions of Bengaluru were selected for this study. A genome-wide association study (GWAS) was performed to identify candidate genes for test-day milk yield, solids-not-fat (SNF), milk lactose, milk density and clinical mastitis. Furthermore, the cross-population extended haplotype homozygosity (XP-EHH) methodology was adopted to scan the dairy cattle breeds (Holstein Friesian, Jersey and Crossbred) in Bengaluru. Two SNPs, rs109340659 and rs41571523, were observed to be significantly associated with test-day milk yield. No significant SNPs were observed for the remaining production traits. The GWAS for milk lactose revealed one SNP (rs41634101) that was very close to the threshold limit, though not significant. The potential candidate genes fibrosin-like 1 (*FBRSL*) and calcium voltage-gated channel auxiliary subunit gamma 3 (*CACN*) were identified to be in close proximity to the SNP identified for test-day milk yield. These genes were observed to be associated with milk production traits based on previous reports. Furthermore, the selection signature analysis revealed a number of regions under selection for the breed-group comparisons (Crossbred-HF, Crossbred-J and HF-J). Functional analysis of these annotated genes under selection indicated pathways and mechanisms involving ubiquitination, cell signaling and immune response. These findings point towards the probable selection of dairy cows in Bengaluru for thermotolerance.

## 1. Introduction

Crossbreeding of Indian dairy cows (descript/native and non-descript) with exotic breeds was introduced to boost milk production through rapid improvement in the native germplasm [1]. Crossbreeding practices that formed the crossbred cattle ‘Taylor’ in Patna, Bihar, were adopted in India as early as 1875 [2]. However, they were implemented on a larger scale in 1963 with the introduction of the Intensive Cattle Development Project (ICDP) as a component of the Special Development Program in the framework of the Third Five-Year Plan [1]. Such crossbreeding programs played a significant role in placing India as the world’s largest milk producer [1]. However, in the absence of a well-enforced breeding policy, further crossing of the F1 progeny usually implies a productivity decline in the F2 and subsequent generations [3]. Furthermore, extensive crossbreeding practices using exotic cattle increase the population size of admixed cattle. Smallholder farmers retain such admixed cattle typically in herds of 1 to 10 cows that are mostly bred by artificial insemination [4]. Moreover, these herds do not have proper pedigree records or individual animal performance, thereby lacking information for further genetic improvement. In such a situation, where genetic improvement by traditional breeding schemes is hampered, genomic approaches could help in understanding genetic mechanisms that could be exploited in practical breeding and selection schemes.

The availability of high-density single-nucleotide polymorphism (SNP) markers has substantially enhanced the genomic selection process [5]. It is now possible to identify genomic regions associated with traits of interest for future selection through genome-wide association studies (GWASs) [5]. This analysis depends on linkage disequilibrium (LD) between SNPs and the causal variants, implying the availability of both genotype and phenotype data for the analysis [5]. The LD between SNP markers and causal variants in an admixed population could be an outcome of the LD passed down from the parental population, also formed when crossing populations [6]. Furthermore, GWASs also contribute to identifying potential candidate genes associated with economic and resilience traits. Several researchers adopted such methodologies to identify potential candidate genes and signatures of selection [7,8].

In recent years, a quite large number of studies focused on identifying selection signatures in livestock and evaluating their potential to detect candidate markers associated with economically important traits. Several statistical approaches have been developed to detect selection signatures, including Tajima’s D-statistic [9], Fay and Wu’s H-statistic [10], Composite of Likelihood Ratio (CLR) [11], extended haplotype homozygosity (EHH) [12], cross-population extended haplotype homozygosity (XP-EHH) [12] and the integrated haplotype score (iHS) [13]. The XP-EHH method is an extended approach of EHH and iHS that compares long haplotypes between populations and identifies selected alleles based on their respective high frequency or fixation within a single population [12]. The added benefit of adopting this methodology is that it represents the recent signatures of selection, thereby harboring genomic changes caused by recent selective pressures that may include performance gain and breed formation [14].

A quite large number of studies worldwide focused on the detection of selection signatures in cattle [15,16,17,18]. However, most of these studies considered commercially available cattle breeds [14,17,18] or created divergent groups according to conventional traits [16,19] by neglecting environmental or demographic characteristics as group stratification criteria. Likewise, a major proportion of such studies were conducted in developed countries having temperate climates, while limited reports address native breeds or crossbred cattle in developing countries kept under harsh environmental conditions. As indicated above, crossbreeding using assisted reproductive techniques (artificial insemination) is a widely practiced strategy in Indian dairy cows. Since predominantly smallholder farmers practice dairy farming in Bengaluru, animals there are mostly subjected to natural and non-systematic artificial selection in ongoing generations. Furthermore, exposure to multiple environmental stressors like harsh climatic conditions, infectious diseases and low- and poor-quality feed contributes to natural instead of artificial selection [15]. Such environmental constraints are motivators for studies exploring the footprints of selection linked to adaptation and production in dairy cattle reared under challenging tropical conditions.

Also, with regard to GWASs, numerous studies have been performed on dairy cattle across the globe, but there is a scarcity of reports addressing the tropical environmental context, especially for cow health traits. In such a context, genes with well-known effects on commercial cow traits in developed countries might be switched off or on, pointing to possible genotype-by-environment interactions. Likewise, to the best of our knowledge, no previous study has focused on both GWASs and selective sweeps in Indian dairy cows reared by smallholder farmers in a harsh tropical environment by applying “alternative” selection strategies over decades.

Consequently, the aims of this study were (i) to perform a GWAS and to estimate SNP-based genetic parameters for milk yield and mil- associated traits, as well as for clinical mastitis, to identify important genomic regions and potential candidate genes associated with variation in these traits and (ii) to compare selection signatures based on XP-EHH considering exotic and crossbred cattle.

## 2. Materials and Methods

### 2.1. Study Location and Phenotypic Trait Recording

The study was conducted in the rising metropolitan city of Bengaluru in southern India, where smallholder dairy farmers rear cattle across the rural–urban interface. A total of 68 farms housing 240 lactating dairy cows with an age from 3 to 6 years were selected for the study. All the selected animals were apparently healthy without any visible signs of disease or infection. The animals reared in this region had favorable hygiene scores with mild variation across the rural–urban interface [20]. The farms were monitored for three seasons, “summer” (March–June), “monsoon” (July–October) and “winter” (November–February) [21,22], for cow trait recording and for genotyping. All the traits were recorded once per season. The phenotypic traits recorded included test-day milk yield, milk composition traits (SNF, milk lactose, milk protein and milk density) and a test for clinical mastitis. Approximately 30 mL of milk per cow was collected to assess the milk composition variables using a Lactoscan Milk Analyzer (Softrosys Technologies, Bengaluru, India). The mastitis test was performed for all four quarters of the udder using the California Mastitis Test (CMT) following the protocol suggested by Kandeel et al. [23]. The overall CMT score of an animal during each recording was determined based on the quarter with the highest CMT score. This was done to generate a data structure representing a quite high infection status and pronounced phenotypic trait variation. The CMT scores of negative, trace, 1, 2 and 3 were numerically recorded as 1, 2, 3, 4 and 5, respectively. The survey stratification index (SSI) was used to distinguish the rural–urban interface into “urban” (SSI < 0.3), “transition” or “peri-urban” or “mixed” (SSI: 0.3–0.5) and “rural” (SSI > 0.5) [24]. The dairy cows were categorized into three genetic groups, crossbred, exotic and native cattle. Holstein Friesian (HF) and Jersey (J) breeds comprised the exotic group of animals, while the crossbred cattle were crosses of Holstein and Jersey with native breeds. The native breeds involved in the study were predominantly Hallikar and a few Khillar cows. For the genomic studies, we focused on comparisons and selection signatures considering the exotic and crossbred cattle. In ongoing studies, additional consideration of pure native breeds might contribute to inferring further sweeps of selection.

The basic animal and farm information included breed, lactation number, lactation stage and SSI. Furthermore, meteorological variables including temperature and humidity were recorded on the farms at hourly intervals using VOLTCRAFT DL-121TH USB data loggers. The temperature humidity index (THI) was calculated using the equation of the National Research Council (NRC) [25]:

THI = (1.8 × T + 32) − (0.55 − 0.0055 × RH) × (1.8 × T − 26)(1)

where T is air temperature in degrees Celsius and RH is the relative humidity in percent.

### 2.2. Genotyping and Quality Control

Genotyping was conducted using the *Illumina Bovine 50K SNP BeadChip V2* (96 cows) and the *Illumina Bovine 62K SNP BeadChip* (144 cows) chips. Animals with low-density genotypes were imputed to 62K, and after imputation, 45,054 SNPs were available from 213 genotyped cows. The SNPs were then processed for quality control using the software package PLINK, version 2.0 [26]. SNPs with allele frequency lower than 0.05 and those located on the sex chromosomes were discarded. All genotyped animals and SNPs had call rates larger than 90% [27,28]. An overall missing genotype value of 6.337% was calculated for the missing rate considering all markers and animals. Finally, 42,199 SNPs from 126 cows were available for the genomic analyses.

### 2.3. Estimation of Genomic Parameters for Milk and Milk Traits

The genetic parameters for test-day milk yield, milk composition and clinical mastitis were estimated using the BLUPF90 software package [29]. The variance components and heritabilities were estimated using the average information restricted maximum likelihood algorithm implemented in the AIREMLF90 program [30]. The observed heterozygosity, expected heterozygosity and genomic inbreeding coefficient for the breeds were calculated using PLINK [29] and the PreGSf90 program [31]. The statistical model for the genetic analyses of test-day milk yield, milk composition and clinical mastitis was as follows:

**y** = **Xb** + **Zg** + **Wpe** + **e** (2)

where **y** is a vector of phenotypes, test-day milk yield, SNF, milk lactose, milk density and clinical mastitis; **b** is a vector of fixed effects including breed (crossbred, exotic), test-day THI (covariate), lactation number (1 (30 cows), 2 (36 cows), 3 (25 cows), 4 (21 cows), 5 (7 cows), >6 (7 cows)), lactation stage (<3 months (50 cows), 3–9 months (47 cows), >9 months (29 cows)) and SSI (rural, mixed, urban); **g** is a vector of random genomic effects, following N(0, Gσ2g) where **G** is a genomic relationship matrix [30] and σ2g is the respective genomic variance; **pe** is a vector for permanent environmental effects; **e** is a vector of random residual effects; and **X, Z** and **W** are incidence matrices for **b**, **g** and **pe**, respectively.

### 2.4. Genome-Wide Associations and Gene Annotations

The GWAS for the milk traits considered the fixed effects from model (2) and was performed using the BLUPF90 program [29]. The threshold level for the genome-wide significance was a Bonferroni-corrected threshold (PBonf = 0.05/N, N = number of SNP markers). Additionally, a less stringent significance threshold (P_ls_) of −log10 (0.00005) was also applied.

The significant SNPs were considered for ongoing candidate gene annotations using the Bos taurus ARSUCD1.2 genome assembly. A window size of 200 kb (100 kb upstream and 100 kb downstream) for a significant SNP was defined to annotate potential candidate genes. The functions of all identified genes were searched for manually based on published literature and public databases.

### 2.5. Selection Signature Analysis

The cross-population extended haplotype homozygosity (XP-EHH) approach [10] was adopted to scan the dairy cattle breeds in Bengaluru for candidate regions under selection. The previously mentioned imputed, filtered and quality-checked genotype data consisting of Crossbred, Holstein Friesian, Jersey and native cow breeds were considered for the analysis. The between-population selection signatures were assessed for the Crossbred_HF, Crossbred_J and HF_J breed groups. The XP-EHH scores were calculated for each pairwise comparison using the rehh package in R. The positive and negative selection signatures were detected based on the XP-EHH values and by setting the threshold of the top 0.1 percent for both tails of the distribution (lower and upper tails). The genes falling with a window size of 200 kb (100 kb upstream and 100 kb downstream) from the potential regions under selection (positive and negative) were annotated using the Bos taurus ARSUCD1.2 genome assembly. Bioinformatics analysis to assess the functional pathways and enrichment was performed using the DAVID database.

## 3. Results

### 3.1. Genomic Heritabilities and Variance Component Estimates

The observed heterozygosity, expected heterozygosity and genomic inbreeding coefficients are depicted in Supplementary Table S1. Table 1 depicts the SNP-based heritabilities and variance component estimates for the production traits and clinical mastitis. The heritabilities for test-day milk yield, SNF, lactose and density were 0.25 ± 0.21, 0.13 ± 0.05, 0.20 ± 0.16 and 0.17 ± 0.23, respectively. Interestingly, among all traits, clinical mastitis had the largest heritability with the smallest stand error of 0.48 ± 0.07.

### 3.2. Genome-Wide Associations and Potential Candidate Genes

**Figure 1:**
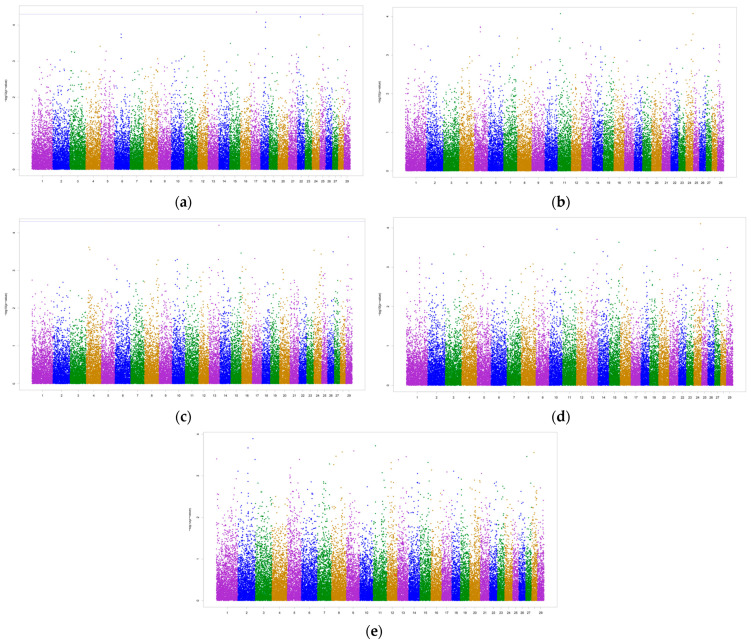
Manhattan plots for test-day milk yield (**a**), solids-not-fat (SNF) (**b**), lactose (**c**), density (**d**) and clinical mastitis (**e**). The blue line is the significance threshold line for the less stringent significance threshold (P_ls_) of −log10 (0.00005), and the dots above this line represent significant SNPs according to a false discovery rate of 5%.

A GWAS was performed to identify the genetic variations associated with the traits of interest, i.e., test-day milk yield, milk SNF, lactose, density and clinical mastitis. The Manhattan plots for these traits are presented in Figure 1.

The GWAS revealed two significant SNPs, rs109340659 on BTA 17 and rs41571523 on BTA 25, above P_ls_ for test-day milk yield. The significant markers were associated with two potential candidate genes, fibrosin-like 1 (*FBRSL*) and calcium voltage-gated channel auxiliary subunit gamma 3 (*CACN*). No significant SNPs were detected for the remaining traits. However, the GWAS for milk lactose revealed one SNP (rs41634101) very close to the significance threshold, though not significant. This SNP, located on BTA 13, was annotated with the genes ubiquitin-conjugating enzyme E2 V1 (*UBE2V1*), transmembrane protein 189 (*TMEM189*), *ENSBTAG00000049867* and CCAAT enhancer-binding protein beta (*CEBPB*).

### 3.3. Selection Signatures

**Figure 2:**
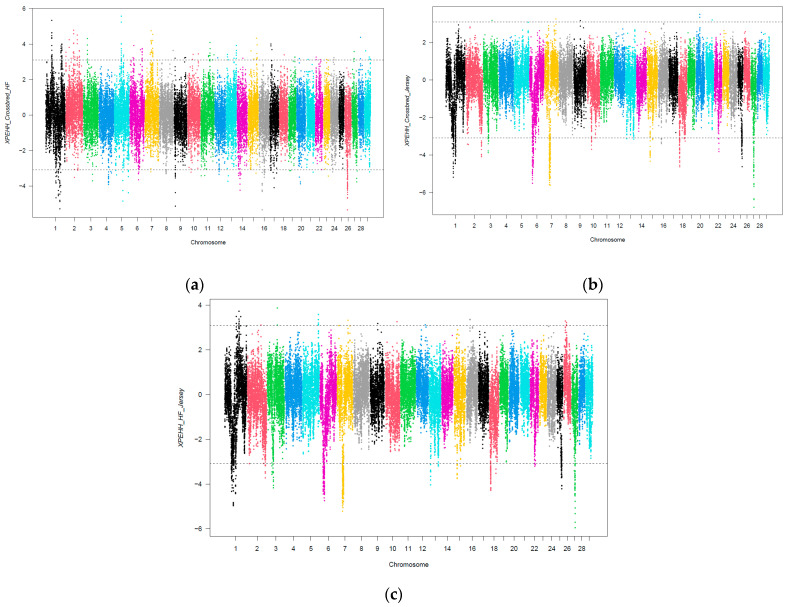
Distribution of XP-EHH values across the genome for Crossbred-HF (**a**), Crossbred-Jersey (**b**) and HF-Jersey (**c**) groups. The *x*-axis depicts the SNP position in the genome, and the y-axis depicts the XP-EHH values. The dotted lines indicate the top 0.1 percentile for positive and negative selection for each comparison.

Figure 2 depicts the Manhattan plots for the standardized XP-EHH score including the comparisons for the Crossbred-HF (Figure 2a; Crossbred: 37 cows; HF: 50 cows), Crossbred-J (Figure 2b; Crossbred: 37 cows; Jersey: 7 cows) and HF-J (Figure 2c; HF: 50 cows; Jersey: 7 cows) dairy cow breed groups.

The negative XP-EHH values reflect selection signatures in Crossbred (for Crossbred-HF and Crossbred-J comparisons) and in HF (for HF-J comparison) populations. Similarly, the positive XP-EHH values reflect selection in the HF (Crossbred-HF comparison) and Jersey (Crossbred-J and HF-J comparisons) breed groups. Based on the XP-EHH scores and the threshold definition (top 0.1 percentile of positive and negative values) 178, 6 and 27 regions were found to be positively selected for the Crossbred-HF, Crossbred-J and HF-J groups, respectively. Similarly, 181, 417 and 459 candidate regions were observed to be negatively selected for the Crossbred-HF, Crossbred-J and HF-J groups, respectively. Further gene annotation analyses revealed 216, 5 and 39 genes to be associated with the identified positive selection sweeps for the Crossbred-HF, Crossbred-J and HF-J groups, respectively. The numbers of annotated genes with regard to negative selection and the same group comparisons were 185, 353 and 340 genes, respectively.

### 3.4. Functional Analysis

**Figure 3:**
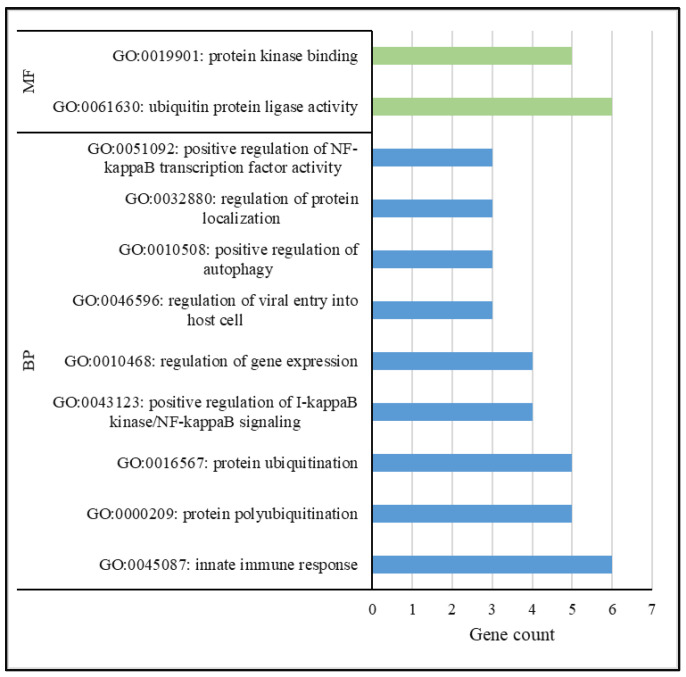
Functional annotation clustering for the gene ontology terms (BP: Biological Process; MF: Molecular Function) of the positive selection sweeps for Crossbred-HF.

Using the default settings in DAVID, the significantly enriched (*p* < 0.05) GO terms and KEGG pathways were assessed. With regard to the genes associated with the positive selection sweeps for each breed comparison, 23, 2 and 9 GO terms were significantly enriched (*p* < 0.05) for Crossbred-HF, Crossbred-J and HF-J comparisons, respectively. The functional annotation clustering in DAVID created a further cluster of similar annotations, contributing to an improved understanding of the functional mechanisms involved. The functional annotation clustering of the positive selection sweeps for the Crossbred-HF comparison revealed a number of pathways associated with immune response. The respective enrichment score was 0.81 (Figure 3).

**Figure 4:**
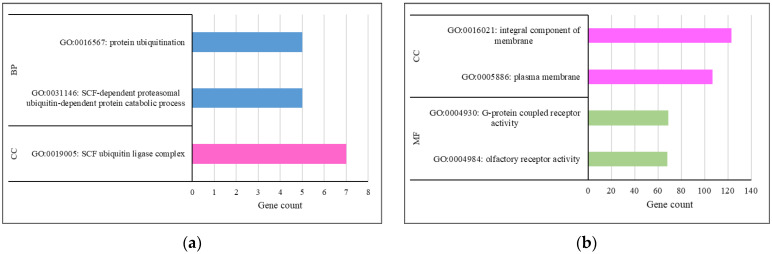

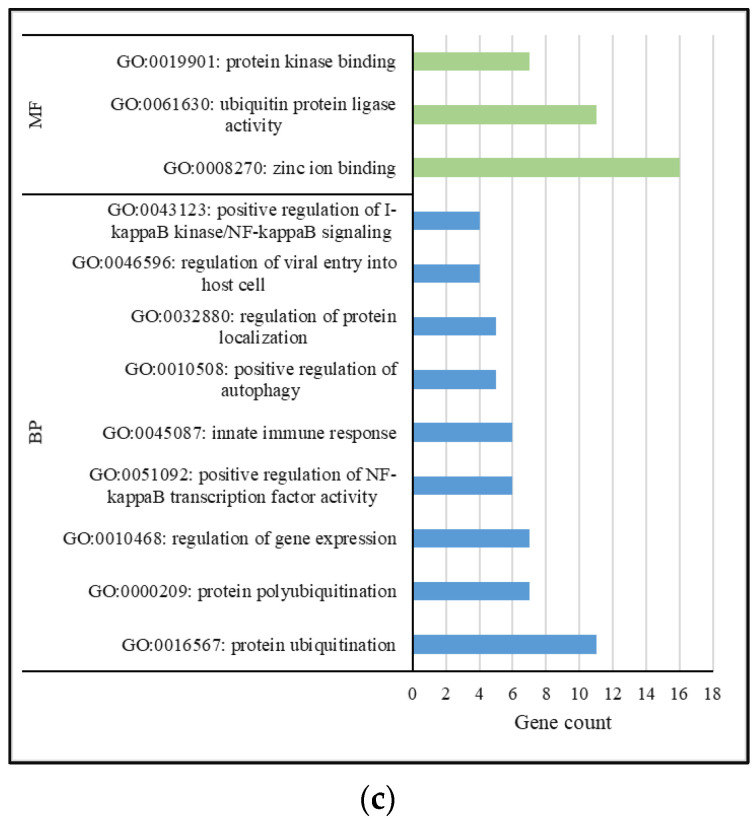
Functional annotation clustering for the gene ontology terms (BP: Biological Process; CC: Cellular Component; MF: Molecular Function) of the negative selection sweeps for Crossbred-HF (**a**), Crossbred-Jersey (**b**) and HF-Jersey (**c**).

Similarly, functional annotations of the genes associated with negative selection sweeps revealed 27, 19 and 22 GO terms for Crossbred-HF, Crossbred-J and HF-J comparisons, respectively. The results for functional annotation clustering of the negative selection sweeps for the Crossbred-HF, Crossbred-J and HF-J comparisons implied several clusters. The identified clusters with the respective enrichment scores of 2.68, 12.26 and 1.44 are depicted in Figure 4.

Further bioinformatics analysis was performed to identify the KEGG pathways associated with the identified genes under selection for the three breed-group comparisons. Functional annotation clustering of the negative selection sweeps for Crossbred-HF depicted enriched KEGG terms (enrichment score: 1.22) including ovarian steroidogenesis (bta04913), prolactin signaling pathway (bta04917), cortisol synthesis and secretion (bta04927), steroid hormone biosynthesis (bta00140) and Cushing syndrome (bta04934) (Supplementary Table S1). Likewise, a number of KEGG terms were identified for the Crossbred-J and HF-J comparisons. These clustering annotations depicted that a few stress-related KEGG terms like oxidative phosphorylation (bta00190), thermogenesis (bta04714) and pathways of neurodegeneration—multiple diseases (bta05022) were enriched both in Crossbred-J and HF-J. A detailed overview of the functional annotation clustering of the KEGG pathways for the negative selection sweeps is given in Supplementary Table S2.

## 4. Discussion

The milk production and composition traits displayed a broad SNP-based heritability range. Though a number of researchers have assessed the heritability estimates for milk yield and milk composition traits in different dairy cattle breeds globally, there are minimal reports based on dense genomic marker data in tropical countries. Furthermore, to the best of our knowledge, this study is the first report addressing genetic and genomic parameter estimations for milk traits in Indian dairy cows. In the present study, test-day milk yield had a moderate heritability of 0.25 ± 0.21. This was similar to the values (0.257 ± 0.063) reported for a Bangladesh native cattle breed [32] and those (0.26 ± 0.11) for US Jersey cows [33]. Generally, milk production traits have a moderate heritability. Only a few studies, mostly considering data from small family farms, have reported low heritability estimates for milk yield of 0.12 (Chinese Holstein cows; [34]), 0.14 (Italian Holstein cows; [35]) and 0.15 (Black Pied cattle; [36]). Higher estimates of 0.35 (German Holstein cattle; [37]), 0.37 (Finnish Ayrshire; [38]) and 0.43 (Finnish Holstein Friesian; [38]) were reported in studies focusing on larger herds with large contemporary groups. The dairy cows in the present dataset are kept in production systems characterized by small herd sizes (ranging from two to eight cows per herd) and minimal investments towards farming system improvements. Furthermore, animals reared on family farms in tropical regions are often exposed to severe environmental stressors [21]. In contrast to most commercial farms and herds in developed countries, the majority of animals in the present dataset lacked intense management and nutritional interventions to enhance production and alleviate stress. Consequently, the genomic heritability for milk yield was similar to estimates from another tropical region, i.e., Bangladesh [32], and also for subsets of populations reflecting heat stress in developed countries such as the US [33]. Furthermore, the comparatively large environmental variance compared to the additive genetic component indicates the challenging environmental conditions that were not captured by the statistical model effects. The inter-herd variations in milk yield due to feeding, breed, husbandry characteristics or cow treatments as identified in phenotypic trait analyses in Bengaluru [21] cannot be covered through the modeling of herd or SSI effects.

The genomic heritability estimate for SNF in this study for the Bengaluru cattle population was 0.13 ± 0.05 and therefore smaller compared to estimates of 0.27 in US Jerseys [33], 0.29 in Japanese Holsteins [39] or 0.36 in other large Jersey populations [40]. Lower heritability estimates for SNF comparable to our results ranging between 0.07 and 0.13 at different days in milk were reported by Cho et al. [41] for Korean Holstein cows. In our study, the heritability for milk lactose was 0.20 ± 0.16. Only a few studies estimated genetic parameters for lactose content because this trait is not considered in official cattle breeding goals. Our genomic heritability was smaller compared to pedigree-based estimates of 0.33 as reported by Tiezzi et al. [42] and Petrini et al. [43] in Holstein cows and by Sneddon et al. [44] in a multi-breed population approach. The heritability for milk density was 0.17. There is a lack of studies estimating the heritability for milk density in dairy cows, but milk density is favorably correlated with SNF [45]. Quite large additive genetic variances for milk density and SNF were reported by Kawahara et al. [39] from pedigree-based analyses. Hence, the selective genotyping in our study might not reflect the full genetic variation in the dairy cattle population from Bengaluru. Also for SNF, milk density and lactose content, the residual variation was stronger than the additive genetic variation. Again, nutritional variation between herds might inflate the residual variance component.

Mastitis is among the most prevalent diseases in dairy cattle worldwide, causing high economic losses [46], as well as in Bengaluru. Heritabilities for clinical mastitis vary widely, from 0.01 to 0.42 [47,48]. In large commercial breeds, genomic and pedigree-based heritabilities were smaller than 0.10 [49,50]. The wide range of heritabilities might be due to the trait definition, the scoring system and the observer influence. In the present study, the quite large heritability of 0.48 ± 0.07 for clinical mastitis may be due to estimates relying on CMT scores and only two well-trained observers being responsible for health trait recording. Accordingly, in a study by Alrawi et al. [46], the heritability for monthly coded CMT scores in US Holstein cows ranged from 0.11 to 0.48. Also, Gonyon et al. [51] determined a high heritability for clinical mastitis (0.23) for Pacific Northwest Holstein cows using the CMT score. The study by Bouyai et al. [52] conducted on tropical Holsteins proved that clinical mastitis recorded as a categorical trait with several scores contributed to increased heritability estimates compared to a binary trait definition. However, in their study, the SE for clinical mastitis with several categories was larger than that for the binary disease. Additionally, the high prevalence of clinical mastitis (32.7%) in the studied population might have contributed to the high CMT score (2.01) and the associated pronounced phenotypic variation. As indicated above, we considered the CMT score from the udder quarter indicating the highest level of infection pressure. Such a strategy contributed to increased CMT values and phenotypic and genetic variations. Furthermore, we recorded the disease indicator CMT under harsh environmental conditions. For the estimation of genetic parameters of disease traits, Wagner et al. [27] suggested considering only records from challenging environments, i.e., herds with a high infection pressure. In such herds, there was an obvious genetic differentiation for udder health among cows, explaining the increased heritabilities. In well-managed herds, almost all cows are scored as healthy, implying a shrinkage of genetic variation.

Regarding the GWAS for milk traits, only two SNPs were significantly associated with milk yield. The quite small number of significant marker associations identified in the current population may be due to the small cow sample size considered for genotyping. Nevertheless, the identified SNPs were annotated with genes with well-known effects on the trait of interest. Such previous reports for candidate genes could be supported by the results for the harsh tropical environment of Bengaluru. The two SNPs identified for milk yield were associated with two potential candidate genes, *FBRSL* and *CACN*. *FBRSL1* plays a vital role in a number of biological processes in mammals, like stem cell maintenance and differentiation [53]. *FBRSL1* was significantly associated with milk yield in Chinese Holstein cows based on a GWAS for haplotypes [7]. In another genome-wide scan conducted on a tropically adapted Indian composite crossbred dairy cattle breed (Vrindavani cattle), the *CACN* gene was significantly associated with milk copper content [8].

The GWAS for milk lactose identified an SNP (rs41634101) close to the threshold of significance. This SNP is located on BTA 13 in a close chromosomal neighborhood to the genes *UBE2V1*, *TMEM189*, *ENSBTAG00000049867* and *CEBPB*. The gene *UBE2V1* codes a protein variant of the ubiquitin-conjugating E2 enzyme, playing a vital role in ubiquitination, a type of posttranslational modification [54]. In a genome-wide profiling study by Rani et al. [55] using microRNA expression in buffalo milk exomes, *UBE2V1* was one of the target genes among the 10 most abundant exosomal microRNAs. *CEBPB*, coding for a protein under the family of CCAAT enhancer-binding proteins, regulates genes associated with proliferation and differentiation in a number of cell types. These groups of proteins play vital roles in the development of the mammary gland and lactation [56,57]. Furthermore, *CEBPB* is essential for the expression of the beta casein milk protein [58]. Therefore, the identified candidate genes are of high relevance for milk production in different countries, and this also seems to be true for the challenging environment of Bengaluru, as indicated by the present results.

Another novel aspect of this study was the assessment of the selection sweeps among the dairy cattle breeds in Bengaluru. The identification of selection signatures in livestock populations might unravel genes and biological mechanisms associated with domestication, breed development and artificial selection [5]. Knowledge of selection signatures could give an inroad towards selection for economically important traits and also for adaptation and tolerance traits. Researchers across the globe have adopted this approach in purebred [59], composite breed [18] and admixed livestock populations [5,60]. The findings of the present study give an overview of the regions under selection in dairy cow breed groups including Holstein Friesian, Jersey and crossbreds in a tropical selection environment. The results obtained in this study are in concordance with findings from gene expression analyses in relation to heat stress in a smaller cattle population from Bengaluru, also highlighting the importance of these genes in immune response mechanisms [22].

With regard to the functional annotation clustering, the Crossbred-HF comparison revealed some interesting GO terms like ubiquitin protein ligase activity (GO:0061630), protein kinase binding (GO:0019901), protein ubiquitination (GO:0016567), innate immune response (GO:0045087) and positive regulation of I-kappaB kinase/NF-kappaB signaling (GO:0043123). Most of these terms were associated with ubiquitination and immune response. Functional annotation clustering confirmed similar GO terms for the negative selection sweeps for Crossbred-HF, Crossbred-J and HF-J comparisons. The majority of the terms were associated with ubiquitination, immune response and cell signaling. These selection footprints support evidence for multiple stress mechanisms in dairy cows in tropical regions. For example, Cheruiyot et al. [15] reported distinct selection sweeps in admixed cattle in Tanzania that were associated with adaptation and productive performance. Ubiquitination plays a crucial role in ensuring protein homeostasis by removing unwanted or damaged proteins [61]. This pathway regulates several basic cellular processes, few among them reflecting environmental stress, immune response and DNA repair mechanisms [62]. The enrichment of these terms is of high relevance for cattle populations in tropical savanna regions with long-lasting heat stress conditions [21]. In further causality, molecular responses to heat stress were associated with the immune response mechanisms of dairy cows [22]. Therefore, the results of the present study based on selective sweeps substantiate such findings.

The KEGG pathways obtained upon functional clustering consisted of different terms. Specifically, it is very interesting to observe a few stress-associated KEGG pathways for the negative selection sweeps in Crossbred-HF, like cortisol synthesis and secretion (bta04927), steroid hormone biosynthesis (bta00140), ovarian steroidogenesis (bta04913) and prolactin signaling pathway (bta04917). The genes associated with these KEGG terms were steroid 17-alpha-hydroxylase/17,20 lyase (*LOC112441470*), luteinizing hormone/choriogonadotropin receptor (*LHCGR*), cytochrome P450 family 17 subfamily A member 1 (*CYP17A1*) and steroid 17-alpha-hydroxylase/17,20 lyase (*LOC112444495*). Similarly, the genes annotated from the negative selection sweeps for Crossbred-J and HF-J depicted some enriched KEGG terms including oxidative phosphorylation (bta00190) and thermogenesis (bta04714) with high relevance for a tropical environment. The genes associated with these terms were ATP synthase F1 subunit delta (*ATP5F1D*), cytochrome c oxidase copper chaperone COX17 (*COX17*), ubiquinol-cytochrome c reductase complex III subunit XI (*UQCR11*), UQCRQ, cytochrome c oxidase subunit 7A1 (*COX7A1*), NADH: ubiquinone oxidoreductase subunit B4 (*NDUFB4*), NADH: ubiquinone oxidoreductase core subunit S7 (*NDUFS7*), and cytochrome c oxidase subunit VIIb (*COX7B*). These genes are common for both breed comparisons Crossbred-J and HF-J and indicate the genetic contribution of Jersey germplasm.

The present study has its uniqueness because it is based on data from smallholder cattle farming systems in India reflecting a challenging tropical environment. The results obtained from this study are of primary relevance for all stakeholders involved in cattle farming. However, we also see some limitations. The quite large number of environmental stressors (SSI, heat stress, husbandry and feeding conditions) complicate the statistical modeling approaches and contribute to enlarged SE. Furthermore, in tropical countries, genotyping and phenotyping remain a challenge, implying limitations in sample sizes. Hence, the quite large SE accompanying genetic and genomic parameter estimates is a common characteristic for studies conducted in developing countries. There are a few reports addressing GWASs and selection signature analyses in Indian cattle populations based on even smaller numbers (24 to 96 cows) [18,63,64]. We are aware of the small sample size. Nevertheless, we identified and verified potential candidate genes in such a small dataset for milk traits that previously had been reported in large-scale studies in commercial breeds, indicating the reliability of the present study. Therefore, the findings from this study stimulate ongoing research in tropical countries, considering genomic mechanisms in the context of environmental challenges.

## 5. Conclusions

This study was the first of its kind aiming at the identification of genomic variants associated with milk production traits and the exploration of selection sweeps in dairy cattle reared under challenging tropical smallholder production systems. Two SNPs, rs109340659 and rs41571523, were significantly associated with test-day milk yield. These SNPs were located in close proximity to the *FBRSL* and *CACN* genes, which were suggested as potential candidate genes for adaptation and productivity under a tropical climate. Using the XP-EHH methodology, several selection sweeps (positive and negative) were identified for the breed group comparisons Crossbred-HF, Crossbred-J and HF-J. Ongoing bioinformatics analyses revealed that these genes were associated with varied stress response mechanisms and adaptation pathways. These functional mechanisms and pathways included ubiquitination, immune response, cell signaling, cortisol synthesis and secretion, the prolactin signaling pathway, steroid hormone biosynthesis and thermogenesis. This study not only provides an insight into the genetic association towards milk production traits, but also gives an insight into the possible evolvement/selection of dairy cattle breeds in Bengaluru for better thermotolerance in a harsh environment. Such findings are of great relevance for researchers and policymakers to initiate ongoing studies in India or in other tropical countries, i.e., to understand mechanisms of selection and genomics in the context of environmental alterations. As the study is limited by a comparatively small sample size, it is of utmost importance to further validate the results in ongoing genomic analyses in tropical breeds reared under stressful conditions.

## Figures and Tables

**Table 1:** Number of genotyped animals; number of records; heritabilities (h^2^); and additive genetic (σ^2^_g_), permanent environmental (σ^2^_pe_) and residual (σ^2^_e_) variances with corresponding SEs (in parentheses).

Trait	Animals	Records (*n*)	h^2^	σ^2^_g_	σ^2^_pe_	σ^2^_e_
Test-day milk yield	125	527	0.25 (0.21)	4.24 (3.59)	5.54 (3.45)	6.92 (0.49)
SNF	126	496	0.13 (0.05)	0.07 (0.03)	0.15 × 10^−4^ (0.46 × 10^−3^)	0.48 (0.04)
Lactose	126	524	0.20 (0.16)	0.024 (0.02)	0.001 (0.02)	0.096 (0.0068)
Density	126	496	0.17 (0.23)	1.42 (1.90)	2.31 (1.89)	4.40 (0.33)
Clinical mastitis	126	267	0.48 (0.07)	0.84 (0.18)	0.13 × 10^−4^ (0.11 × 10^−2^)	0.93 (0.11)

Records (*n*): number of records; SNF: solids-not-fat.

## Data Availability

The anonymized dataset that forms the basis of this article is available through the institutional repository at the University of Göttingen. For scientific purposes, access will be provided upon written request to the corresponding author.

## Supplementary Materials

The following supporting information can be downloaded at: https://www.mdpi.com/article/10.3390/biology12121483/s1, Table S1: Observed heterozygosity, expected heterozygosity and inbreeding coefficient for exotic and crossbred cattle in the selected population.; Table S2: The Kyoto Encyclopedia of Genes and Genomes (KEGG) pathways obtained based on functional annotation clustering using DAVID for the negative selection sweeps of Crossbred-HF, Crossbred-J and HF-J breed group comparisons.
