# Supplementary material for: Unraveling the Genomic Association for Milk Production Traits and Signatures of Selection of Cattle in a Harsh Tropical Environment
Source: Biology (Basel). 2023 Dec 2;12(12):1483. doi: 10.3390/biology12121483 (PMC10740459; doi:10.3390/biology12121483)
Supplement: Supplementary file 1 [file biology-12-01483-s001.zip › biology-2655873-supplementary.pdf]

Supplementary Table S1. Observed heterozygosity, expected heterozygosity and inbreeding coefficient for exotic and crossbred cattle in the selected population.

Breed	Observed Heterozygosity	Expected Heterozygosity	Inbreeding coefficient
**Crossbred**	0.3566	0.3507	0.6187
**Exotic**	0.3533	0.3504	0.5613

Supplementary Table S2. The Kyoto Encyclopedia of Genes and Genomes (KEGG) pathways obtained based on functional annotation clustering using DAVID for the negative selection sweeps of Crossbred-HF, Crossbred-J and HF-J breed group comparisons.

KEGG Term	Gene Count	Raw *p*- Value	Fold Enrichment
**Crossbred-HF (Enrichment Score: 1.20)**			
bta04913: Ovarian steroidogenesis	4	0.02	7.58
bta04917: Prolactin signaling pathway	4	0.03	5.68
bta04927: Cortisol synthesis and secretion	3	0.11	5.34
bta00140: Steroid hormone biosynthesis	3	0.13	4.65
bta04934: Cushing syndrome	4	0.14	3.02
**Crossbred-J (Enrichment Score: 1.11)**			
bta05016: Huntington disease	13	0.02	2.18
bta00190: Oxidative phosphorylation	8	0.02	2.99
bta05415: Diabetic cardiomyopathy	10	0.02	2.49
bta05020: Prion disease	11	0.04	2.09
bta05010: Alzheimer disease	13	0.07	1.73
bta05208: Chemical carcinogenesis—reactive oxygen species	9	0.08	2.01
bta04932: Non-alcoholic fatty liver disease	7	0.09	2.28
bta05012: Parkinson disease	9	0.15	1.71
bta04714: Thermogenesis	8	0.16	1.78
bta04260: Cardiac muscle contraction	4	0.25	2.30
bta05014: Amyotrophic lateral sclerosis	10	0.28	1.40
bta05022: Pathways of neurodegeneration—multiple diseases	12	0.30	1.31
**HF-J (Enrichment Score: 0.86)**			
bta05020: Prion disease	10	0.05	2.08
bta05415: Diabetic cardiomyopathy	8	0.07	2.18
bta05010: Alzheimer disease	12	0.08	1.75
bta05016: Huntington disease	10	0.09	1.83
bta00190: Oxidative phosphorylation	6	0.10	2.46
bta05208: Chemical carcinogenesis—reactive oxygen species	8	0.11	1.95
bta04932: Non-alcoholic fatty liver disease	6	0.15	2.14
bta05014: Amyotrophic lateral sclerosis	10	0.20	1.53
bta04260: Cardiac muscle contraction	4	0.21	2.52
bta05022: Pathways of neurodegeneration—multiple diseases	12	0.21	1.43
bta05012: Parkinson disease	7	0.35	1.45
bta04714: Thermogenesis	6	0.39	1.46
